# Distribution and factors associated with refractive errors among young adults attending ophthalmic clinics in Iraq: a multicenter cross-sectional study

**DOI:** 10.3389/fopht.2026.1904325

**Published:** 2026-09-28

**Authors:** Hassan A. Aljaberi, Saeed Rahmani, Ali Ayatollahi

**Affiliations:** 1 Optical Techniques Department, College of Health and Medical Techniques, Al-Mustaqbal University, Babylon, Iraq; 2 Department of Optometry Techniques, College of Health and Medical Techniques, Al-Furat Al-Awsat Technical University, Najaf, Iraq; 3 Department of Optometry, School of Rehabilitation, Shahid Beheshti University of Medical Sciences, Tehran, Iran; 4 International Campus, Shahid Beheshti University of Medical Sciences, Tehran, Iran

**Keywords:** astigmatism, clinic-based study, Iraq, myopia, refractive errors, young adults

## Abstract

**Objectives:**

To describe the clinic-based distribution of myopia, hyperopia, and astigmatism among young Iraqi adults and to examine demographic, social, familial, and self-reported behavioral factors associated with myopia and high-severity refractive errors.

**Materials and methods:**

This multicenter, clinic-based, cross-sectional study included 4,340 consecutive young adult attendees at three tertiary ophthalmic centers. Right-eye sphere and cylinder values were standardized to minus-cylinder notation before spherical equivalent (SE), and refractive outcomes were derived. Subgroup percentages were calculated using the relevant subgroup denominator, and multivariable logistic regression was used to evaluate associated factors.

**Results:**

In the analytic sample, myopia (SE ≤ −0.50 D) occurred in 51.2%, hyperopia (SE ≥ +0.50 D) in 37.0%, and astigmatism (|cylinder| ≥ 0.50 D) in 66.4%; astigmatism overlapped with spherical refractive status. Myopia was more frequent among women than men (53.6% vs 45.6%). In the adjusted model, female sex, age 28–32 years, a positive family history of refractive error, sleep duration ≤6 hours, and longer daily electronic-device use were associated with higher odds of myopia, whereas office work, manual work, and unemployment were associated with lower odds than student status.

**Conclusions:**

Refractive errors were frequent among young adults attending tertiary ophthalmic clinics in Iraq. The observed associations with demographic, familial, social, and behavioral factors provide clinically useful information for targeted refractive assessment, counseling, and service planning in young adults. Population-based longitudinal studies are needed to confirm the generalizability and temporal nature of these associations.

## Introduction

Refractive errors (REs) are common causes of correctable visual impairment. Myopia, hyperopia, and astigmatism arise from different combinations of axial length, corneal curvature, and crystalline-lens power and may affect visual function, educational performance, and work-related tasks when uncorrected (1–6).

The global burden of myopia and high myopia is projected to increase substantially, and high myopia is associated with myopic maculopathy, retinal detachment, glaucoma, and other causes of irreversible visual loss (7–11). Contemporary evidence emphasizes an interaction between genetic susceptibility and environmental exposure rather than a single causal pathway (12–14).

Urban residence, intensive education, near work, digital-device use, limited outdoor time, and sleep characteristics have been associated with refractive status, particularly myopia (15–17). Because much of this evidence is observational, these variables should be described as correlates or exposures rather than established causes.

Refractive-error estimates vary markedly across regions due to multiple factors, such as differences in age, sampling frame, cycloplegia, and classification thresholds. Studies from the Middle East report substantial heterogeneity, while investigations from Asia, Europe, and China likewise demonstrate age- and setting-specific patterns (18–22).

Young adults are a relevant group for refractive research because refractive change may continue after adolescence and because educational and occupational visual demands are high. However, cross-sectional differences between age groups cannot demonstrate individual progression and should be interpreted as between-group patterns only.

Clinic-based studies can characterize service users and generate hypotheses about associated factors, but they do not estimate community prevalence unless the sampling frame is population based. This distinction is especially important when clinic attendance differs by sex, residence, occupation, symptoms, referral, or access to care.

In Iraq, recent evidence has focused mainly on schoolchildren and selected local samples (23–26). Large multicenter data on young adults are limited, particularly for detailed refractive profiles, severity, and associated demographic, familial, social, and behavioral characteristics. More recently, a multicenter Iraqi pediatric clinic-based study demonstrated distinct refractive profiles across age and strabismus subgroups and emphasized that distributions observed in tertiary clinical samples should not be interpreted as population prevalence estimates (27).

Accordingly, this study aimed to describe the clinic-based distribution of myopia, hyperopia, astigmatism, and mutually exclusive principal-meridian refractive profiles among young adults attending three tertiary ophthalmic clinics in Iraq, and to examine factors associated with myopia and high-severity refractive errors. The study was not designed to estimate national prevalence or establish causal determinants.

## Materials and methods

### Study design and setting

This multicenter, clinic-based cross-sectional, analytical study was conducted at three major tertiary ophthalmic centers in Iraq: Ibn Al-Haitham Teaching Eye Hospital in Baghdad, Imam Hussain Specialized Eye Complex in Karbala, and Al-Hakeem Teaching Hospital in Najaf. All three centers are located in urban areas and provide tertiary ophthalmic services to patients residing in both urban and rural areas within their respective governorates. As the study was based on existing clinical records rather than prospective enrollment, residence status was ascertained from the documented place of residence and categorized as urban or rural. To ensure consistency across sites, a unified examination protocol and a standardized data-collection form were applied at all three centers.

### Study population and recruitment

Consecutive young-adult clinic attendees in four prespecified age groups (18–22, 23–27, 28–32, and 33–37 years) were enrolled from February 2024 to February 2026. The clinic-based design was selected to characterize the case mix and associated factors among users of tertiary refractive and optometric services; the study was cross-sectional and did not include prospective follow-up. Only right-eye data were analyzed to maintain a single eye per participant.

### Inclusion and exclusion criteria

Participants were eligible if they belonged to one of the prespecified age groups and had complete right-eye refraction. Exclusion criteria were a history of corneal refractive surgery, corneal ectasia, significant corneal scarring, media opacity, retinal or optic-nerve disease, ocular trauma, intraocular surgery, or incomplete demographic or refractive information. The source dataset did not include a prescreening log; therefore, the number assessed before construction of the analytic file and the reasons for non-inclusion could not be reconstructed. The final study sample comprised 4,340 eligible participants with complete demographic and right-eye refractive data.

### Data collection and study variables

Demographic variables were age group and sex. Prespecified social variables were governorate, residence (urban/rural), and work or study status (student, office work, manual work, or unemployed). These variables were treated as contextual proxies rather than direct causal exposures. Behavioral variables included self-reported sleep duration (>6 or ≤6 hours/night), primary electronic device type, and average daily electronic device use (1–4, 5–8, or >8 hours/day). Family history was defined as a self-reported history of any refractive error in a first-degree relative. Body mass index, height, weight, and continuous age were not included in the final inferential models because source-data consistency checks indicated that these fields required verification against the original records.

### Ocular examination and refractive assessment

Right-eye uncorrected distance visual acuity was measured with standardized logMAR charts under consistent illumination. Objective refraction was obtained with distance-fixation autorefraction and refined by trained optometrists or ophthalmologists using fogging, monocular best-sphere determination, Jackson cross-cylinder refinement, and binocular balance. The source dataset contained a mixture of plus- and minus-cylinder notation. All records were therefore transposed to minus-cylinder form before the spherical equivalent was calculated as sphere + 0.5 × cylinder.

Cycloplegic refraction was not routinely available in this clinic-based adult assessment. Although non-cycloplegic refraction is commonly used in adult clinical practice, residual accommodation can shift measurements toward myopia and mask hyperopia, particularly in younger adults. The findings should therefore be interpreted as non-cycloplegic clinical refraction, and the absence of cycloplegia is acknowledged as a limitation (12, 28).

### Definitions of refractive errors

Primary spherical refractive status was classified as myopia (SE ≤ −0.50 D), emmetropic SE (−0.49 to +0.49 D), or hyperopia (SE ≥ +0.50 D). Astigmatism was defined independently as cylinder magnitude ≥0.50 D and therefore overlapped with myopia or hyperopia. These definitions align with standard clinical descriptions of refractive errors and the global public health framing of refractive status (29, 30). For additional clinical description, mutually exclusive principal-meridian profiles were derived after minus-cylinder transposition: pure myopia, pure hyperopia, simple myopic astigmatism, simple hyperopic astigmatism, compound myopic astigmatism, compound hyperopic astigmatism, and mixed astigmatism.

### Severity classification of refractive errors

High myopia was defined as SE ≤ −6.00 D, high hyperopia as SE ≥ +5.00 D, and high astigmatism as cylinder magnitude ≥2.00 D. High-myopia and high-hyperopia models were restricted to participants with myopia and hyperopia, respectively; the high-astigmatism model was restricted to participants with astigmatism. These thresholds are widely used in refractive epidemiology, and the definitions of myopia severity align with the International Myopia Institute’s recommendations (19, 30).

### Correction modalities

Current correction modality among included participants was recorded as spectacles or contact lenses.

### Statistical analysis

All final analyses were independently computed from the supplied source dataset using IBM SPSS Statistics version 28.0. Categorical variables were summarized as counts and percentages. Percentages comparing sex or age groups used the relevant subgroup as the denominator. Pearson chi-square tests evaluated categorical distributions; continuity-corrected tests were used for binary comparisons where appropriate. Refractive categories were derived from standardized sphere and cylinder values rather than from the original text labels.

The primary multivariable binary logistic regression modeled myopia (SE ≤ −0.50 D) versus no myopia in the refractive-analysis sample (n = 4,340). Separate models evaluated high myopia among participants with myopia (n = 2,221; 565 events), high hyperopia among participants with hyperopia (n = 1,604; 107 events), and high astigmatism among participants with astigmatism (n = 2,882; 1,371 events). Models included sex, age group, center/governorate, residence, work status, family history, sleep duration, primary screen type, and daily electronic-device use. Adjusted odds ratios (aORs), 95% confidence intervals (CIs), and p-values were reported. Variance inflation factors, receiver operating characteristic area under the curve (AUC), and Hosmer–Lemeshow calibration tests were used to evaluate collinearity, discrimination, and calibration. Statistical significance was set at p < 0.05. Because the severity-specific models were exploratory, p-values were not adjusted for multiple comparisons and should be interpreted cautiously.

### Ethical considerations

The study complied with the Declaration of Helsinki. Ethical approval was obtained from the institutional review boards of Ibn Al-Haitham Teaching Eye Hospital (IRB/IAH/2025-098), Imam Hussain Specialized Eye Complex (IRB/IHC/2025-095), and Al-Hakeem Teaching Hospital (IRB/HTH/2025-089). Written informed consent was obtained from all participants; no consent waiver was used. The analytical dataset was anonymized before analysis.

## Results

### Characteristics of the study population

The final study sample comprised 4,340 participants. Women comprised 70.0% (3,036/4,340) of the analytic sample. The age-group distribution was similar between women and men (Pearson χ² = 0.87, df = 3; p = 0.833). The largest age group was 18–22 years (42.1%), 75.3% lived in urban areas, and 42.9% were students (**Table 1**).

**Table 1:** Characteristics of the analytic study sample (N = 4,340).

Variable	Category	n (%)
Sex	Female	3,036 (70.0)
	Male	1,304 (30.0)
Age group (years)	18–22	1,829 (42.1)
	23–27	1,086 (25.0)
	28–32	867 (20.0)
	33–37	558 (12.9)
Governorate	Baghdad	2,035 (46.9)
	Najaf	1,435 (33.1)
	Karbala	870 (20.0)
Residence	Urban	3,267 (75.3)
	Rural	1,073 (24.7)
Work status	Student	1,860 (42.9)
	Office work	1,541 (35.5)
	Manual work	588 (13.5)
	Unemployed	351 (8.1)
First-degree family history of refractive error	Yes	2,307 (53.2)
	No	2,033 (46.8)
Sleep duration	≤6 hours	2,071 (47.7)
	>6 hours	2,269 (52.3)
Primary screen type	Smartphone	2,160 (49.8)
	Laptop	1,484 (34.2)
	Tablet	696 (16.0)
Electronic device use	1–4 h/day	1,429 (32.9)
	5–8 h/day	1,522 (35.1)
	>8 h/day	1,389 (32.0)
Current refractive correction	Spectacles	3,708 (85.4)
	Contact lenses	632 (14.6)

Percentages use N = 4,340 as the denominator. Age was summarized by the prespecified age-group variable; BMI was not included because of internal inconsistencies in the source file.

### Distribution of refractive outcomes and profiles

Among the 4,340 participants, 51.2% (2,221/4,340) had myopia, 37.0% (1,604/4,340) had hyperopia, and 11.9% (515/4,340) had an emmetropic spherical equivalent. Astigmatism, defined independently by cylindrical power, was present in 66.4% (2,882/4,340) and could overlap with spherical refractive status. Although 515 participants had an emmetropic spherical equivalent, none met the combined definition of no refractive error because all had astigmatism of ≥0.50 D. High myopia, high hyperopia, and high astigmatism occurred in 13.0%, 2.5%, and 31.6% of the sample, respectively. Among the mutually exclusive principal-meridian profiles, pure myopia was the most frequent (22.9%), followed by simple hyperopic astigmatism (16.1%), simple myopic astigmatism (14.9%), and compound myopic astigmatism (14.0%) (**Table 2**).

**Table 2:** Refractive outcomes and mutually exclusive principal-meridian profiles overall and by sex (N = 4,340).

Outcome/profile	Overall n (%)	Female n (%)	Male n (%)	p-value
Overlapping refractive outcomes				
Emmetropic spherical equivalent (−0.49 to +0.49 D)	515 (11.9)	393 (12.9)	122 (9.4)	<0.001
Myopia (SE ≤ −0.50 D)	2,221 (51.2)	1,626 (53.6)	595 (45.6)	<0.001
Hyperopia (SE ≥ +0.50 D)	1,604 (37.0)	1,017 (33.5)	587 (45.0)	<0.001
Astigmatism (|C| ≥ 0.50 D)	2,882 (66.4)	2,049 (67.5)	833 (63.9)	0.023
High myopia (SE ≤ −6.00 D)	565 (13.0)	441 (14.5)	124 (9.5)	<0.001
High hyperopia (SE ≥ +5.00 D)	107 (2.5)	56 (1.8)	51 (3.9)	<0.001
High astigmatism (|C| ≥ 2.00 D)	1,371 (31.6)	955 (31.5)	416 (31.9)	0.799
Mutually exclusive refractive profiles				*Global <0.001*
No refractive error	0 (0.0)	0 (0.0)	0 (0.0)	
Pure myopia	993 (22.9)	773 (25.5)	220 (16.9)	
Simple myopic astigmatism	645 (14.9)	460 (15.2)	185 (14.2)	
Compound myopic astigmatism	608 (14.0)	423 (13.9)	185 (14.2)	
Pure hyperopia	465 (10.7)	214 (7.0)	251 (19.2)	
Simple hyperopic astigmatism	697 (16.1)	505 (16.6)	192 (14.7)	
Compound hyperopic astigmatism	474 (10.9)	333 (11.0)	141 (10.8)	
Mixed astigmatism	458 (10.6)	328 (10.8)	130 (10.0)	

Female and male percentages use sex-specific denominators (female, n = 3,036; male, n = 1,304). Binary p-values are chi-square tests with continuity correction; the profile p-value is the global Pearson chi-square test. Myopia, hyperopia, and emmetropic spherical equivalent were defined by SE, whereas astigmatism was defined independently by cylindrical power; therefore, these outcomes may overlap and should not be summed. No refractive error was defined as an emmetropic SE (−0.49 to +0.49 D) with cylinder magnitude <0.50 D. Although 515 participants (11.9%) had an emmetropic SE, all had astigmatism ≥0.50 D; therefore, none met the combined definition of no refractive error. The seven principal-meridian profiles are mutually exclusive and sum to 100%. SE, spherical equivalent; C, cylinder.

Using sex-specific denominators, myopia was more frequent among women than men (53.6% vs 45.6%), whereas hyperopia was less frequent among women (33.5% vs 45.0%). Astigmatism occurred in 67.5% of women and 63.9% of men. Across age groups, myopia ranged from 48.9% to 52.8%, hyperopia from 35.5% to 38.9%, and astigmatism from 64.3% to 68.0%, without a monotonic age-related pattern (**Table 2**; **Figure 1**).

**Figure 1:**
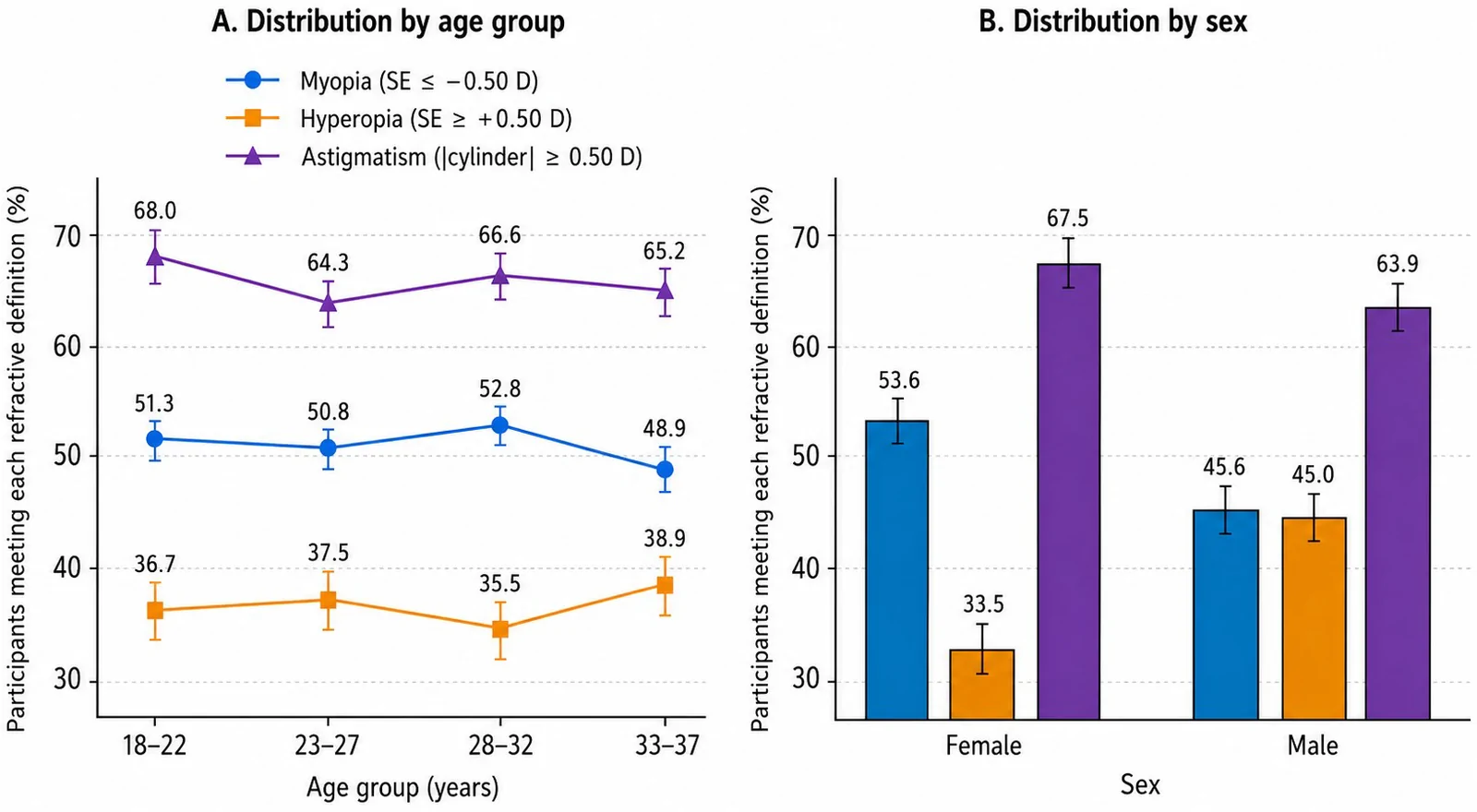
Distribution of refractive outcomes by age group and sex in the analytic cohort (n = 4,340). **(A)** Distribution by age group (18–22, 23–27, 28–32, and 33–37 years). **(B)** Distribution by sex (female and male). Myopia and hyperopia were defined using spherical equivalent, whereas astigmatism was defined using cylindrical power; these categories overlap. Error bars indicate 95% Wilson confidence intervals.

### Unadjusted and adjusted associations with myopia

In unadjusted comparisons, myopia was more frequent among women, participants with a positive first-degree family history of refractive error, those reporting sleep duration ≤6 hours, and those reporting longer daily electronic-device use. Primary screen type was not materially associated with myopia. Because these exposures were self-reported and measured concurrently with refractive status, they were treated as correlates rather than causes.

In the adjusted myopia model (n = 4,340), female sex (aOR = 1.32, 95% CI 1.16–1.51), age 28–32 versus 18–22 years (aOR = 1.28, 95% CI 1.06–1.55), a positive first-degree family history of refractive error (aOR = 1.46, 95% CI 1.29–1.66), sleep duration ≤6 hours (aOR = 1.73, 95% CI 1.53–1.96), and electronic-device use of 5–8 hours/day (aOR = 1.30, 95% CI 1.12–1.50) or >8 hours/day (aOR = 1.80, 95% CI 1.54–2.09) were associated with higher odds of myopia. Office work, manual work, and unemployment were associated with lower odds than student status. The model AUC was 0.632, the Hosmer–Lemeshow p-value was 0.078, and no problematic multicollinearity was identified (**Table 3**; **Figure 2**).

**Table 3:** Multivariable logistic regression for factors associated with myopia (N = 4,340).

Predictor	Comparison	Adjusted OR	95% CI	p-value
Sex	Female vs male	1.32	1.16–1.51	<0.001
Age group	23–27 vs 18–22 years	1.03	0.88–1.21	0.687
	28–32 vs 18–22 years	1.28	1.06–1.55	0.010
	33–37 vs 18–22 years	1.06	0.86–1.32	0.565
Governorate	Najaf vs Baghdad	1.11	0.97–1.28	0.140
	Karbala vs Baghdad	0.97	0.82–1.14	0.708
Residence	Rural vs urban	1.02	0.88–1.17	0.831
Work status	Office work vs student	0.73	0.63–0.85	<0.001
	Manual work vs student	0.77	0.62–0.95	0.015
	Unemployed vs student	0.59	0.45–0.76	<0.001
Family history	Yes vs no	1.46	1.29–1.66	<0.001
Sleep duration	≤6 vs >6 hours	1.73	1.53–1.96	<0.001
Primary screen	Laptop vs smartphone	1.09	0.95–1.25	0.233
	Tablet vs smartphone	1.11	0.93–1.33	0.234
Device use	5–8 vs 1–4 h/day	1.30	1.12–1.50	<0.001
	>8 vs 1–4 h/day	1.80	1.54–2.09	<0.001

OR, odds ratio; CI, confidence interval. The model included all variables shown simultaneously. Reference categories were male sex, age 18–22 years, Baghdad, urban residence, student status, no first-degree family history, sleep >6 hours, smartphone use, and 1–4 h/day of electronic-device use. Model discrimination: AUC = 0.632. Hosmer–Lemeshow goodness-of-fit p = 0.078. Maximum variance inflation factor = 1.50.

**Figure 2:**
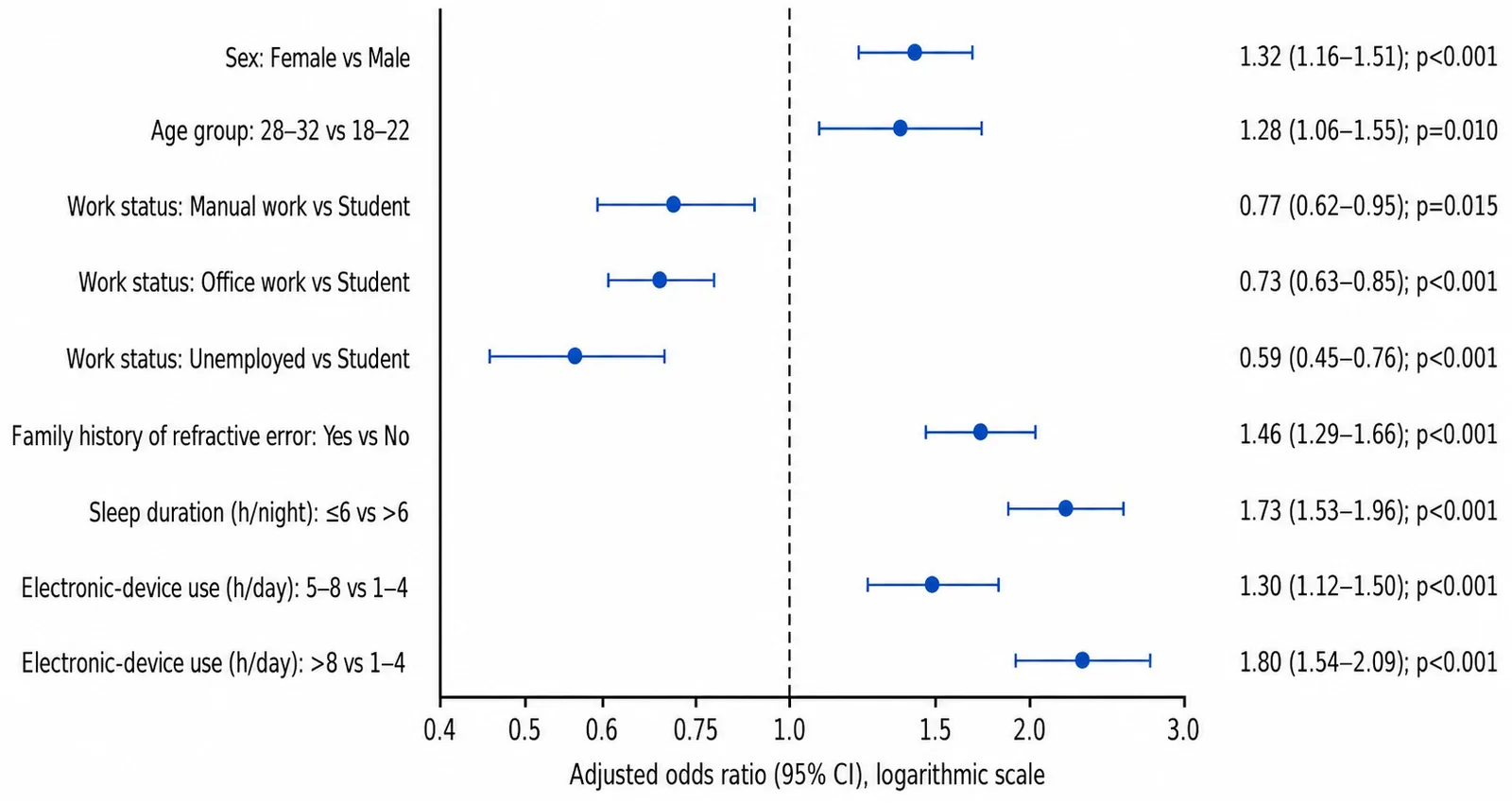
Adjusted associations with myopia in the analytic cohort. Only statistically significant predictors from the full multivariable model are displayed; the complete model is presented in **Table 3**. Points represent adjusted odds ratios and horizontal bars represent 95% confidence intervals. The dashed vertical line indicates an odds ratio of 1.0.

### Factors associated with high-severity refractive errors

Among participants with myopia, high myopia was associated with female sex (aOR = 1.40, 95% CI 1.11–1.76) and was less frequent among rural residents (aOR = 0.57, 95% CI 0.45–0.73) and primary tablet users (aOR = 0.74, 95% CI 0.55–0.98). Among participants with hyperopia, high hyperopia was less frequent among women (aOR = 0.60, 95% CI 0.40–0.90) and those aged 28–32 years (aOR = 0.37, 95% CI 0.18–0.76), but more frequent in Karbala, manual workers, unemployed participants, and those reporting >8 hours/day of device use. Among participants with astigmatism, high astigmatism was less frequent among rural residents (aOR = 0.49, 95% CI 0.41–0.59) and was associated with sleep duration ≤6 hours (aOR = 2.83, 95% CI 2.41–3.31) and electronic-device use of 5–8 hours/day (aOR = 1.50, 95% CI 1.24–1.80) or >8 hours/day (aOR = 3.07, 95% CI 2.52–3.75). AUC values were 0.593, 0.667, and 0.706 for the high-myopia, high-hyperopia, and high-astigmatism models, respectively (**Table 4**).

**Table 4:** Multivariable logistic regression for factors associated with high-severity refractive errors.

Predictor	Comparison	High myopia Adjusted OR (95% CI); p	High hyperopia Adjusted OR (95% CI); p	High astigmatism Adjusted OR (95% CI); p
Sex	Female vs male	1.40 (1.11–1.76); p=0.004	0.60 (0.40–0.90); p=0.014	0.87 (0.73–1.03); p=0.109
Age group	23–27 vs 18–22 years	1.10 (0.86–1.40); p=0.463	0.68 (0.39–1.18); p=0.168	1.00 (0.81–1.23); p=0.991
	28–32 vs 18–22 years	1.18 (0.90–1.56); p=0.235	0.37 (0.18–0.76); p=0.007	1.19 (0.91–1.56); p=0.208
	33–37 vs 18–22 years	1.14 (0.82–1.58); p=0.435	0.66 (0.33–1.32); p=0.241	1.33 (0.98–1.81); p=0.066
Governorate	Najaf vs Baghdad	0.91 (0.73–1.14); p=0.412	1.14 (0.71–1.83); p=0.600	0.96 (0.80–1.15); p=0.636
	Karbala vs Baghdad	0.98 (0.76–1.28); p=0.901	1.74 (1.06–2.86); p=0.028	0.96 (0.78–1.18); p=0.702
Residence	Rural vs urban	0.57 (0.45–0.73); p<0.001	0.60 (0.36–1.03); p=0.062	0.49 (0.41–0.59); p<0.001
Work status	Office work vs student	0.83 (0.66–1.04); p=0.098	1.55 (0.89–2.67); p=0.119	1.08 (0.88–1.32); p=0.485
	Manual work vs student	0.83 (0.61–1.15); p=0.265	4.07 (2.01–8.23); p<0.001	0.90 (0.66–1.23); p=0.522
	Unemployed vs student	0.69 (0.45–1.06); p=0.094	4.11 (1.93–8.73); p<0.001	1.03 (0.73–1.47); p=0.859
Family history	Yes vs no	1.17 (0.96–1.43); p=0.112	0.76 (0.51–1.14); p=0.187	1.08 (0.92–1.27); p=0.323
Sleep duration	≤6 vs >6 hours	0.94 (0.77–1.14); p=0.545	0.97 (0.64–1.45); p=0.867	2.83 (2.41–3.31); p<0.001
Primary screen	Laptop vs smartphone	0.97 (0.79–1.20); p=0.797	0.74 (0.46–1.18); p=0.208	1.11 (0.93–1.33); p=0.230
	Tablet vs smartphone	0.74 (0.55–0.98); p=0.036	1.38 (0.80–2.36); p=0.248	0.89 (0.71–1.11); p=0.297
Device use	5–8 vs 1–4 h/day	1.16 (0.90–1.49); p=0.243	1.30 (0.79–2.14); p=0.300	1.50 (1.24–1.80); p<0.001
	>8 vs 1–4 h/day	1.13 (0.88–1.45); p=0.335	1.71 (1.04–2.84); p=0.036	3.07 (2.52–3.75); p<0.001

OR, odds ratio; CI, confidence interval. The high-myopia model included participants with myopia (n = 2,221; 565 high-myopia events); the high-hyperopia model included participants with hyperopia (n = 1,604; 107 high-hyperopia events); and the high-astigmatism model included participants with astigmatism (n = 2,882; 1,371 high-astigmatism events). Each model included the same covariates and reference categories as **Table 3**. AUC values were 0.593, 0.667, and 0.706, respectively. Maximum VIF was <2.0 in all models.

## Discussion

In this multicenter study of young Iraqi adults, refractive errors were highly prevalent, with astigmatism the most common, followed by myopia and hyperopia. The predominance of astigmatism in this cohort likely reflects the high proportion of compound and mixed refractive profiles commonly encountered among young adults in ophthalmic clinical settings. Similar refractive distributions have been reported in university-based and regional studies conducted in the Middle East and comparable populations, indicating that the refractive patterns observed in Iraq align with broader regional trends (18, 31). However, myopia and hyperopia were defined using spherical equivalent, whereas astigmatism was defined independently using cylinder magnitude; consequently, these outcomes overlap and should not be interpreted as mutually exclusive categories that collectively sum to 100%. The absence of participants meeting the combined definition of no refractive error is consistent with the clinic-based nature of the sample and highlights the importance of distinguishing emmetropic spherical equivalent from the absence of refractive error.

Age-group differences were modest and non-monotonic. Myopia frequencies ranged from 48.9% to 52.8% across the four prespecified age groups, and only the 28–32-year group had higher adjusted odds of myopia than the 18–22-year reference group; the other age-group comparisons were not statistically significant. Longitudinal evidence indicates that myopia onset and progression can continue in young adulthood (8, 10, 32); however, the present age-group findings represent cross-sectional differences rather than individual refractive progression (8, 11).

A notable finding was the sex-specific distribution of refractive outcomes. Myopia was more frequent among women, whereas hyperopia was more frequent among men. Similar sex-related differences have been documented in population-based and regional studies, where female sex has been associated with higher myopia frequency (20–22). Behavioral explanations proposed in previous research include longer study duration, increased near-work intensity, and greater exposure to digital devices (17, 33). Biological differences in ocular growth and refractive development may also contribute (34). Sex-specific denominators were used, and female sex remained associated with myopia after multivariable adjustment. Educational intensity, outdoor exposure, cumulative near-work activity, and sex-related patterns of care-seeking were not measured directly; the association should therefore be interpreted as statistical rather than as evidence of a direct biological effect.

Work or study status also showed an independent association with myopia. Compared with students, participants engaged in office work, manual work, and those who were unemployed had lower adjusted odds of myopia. Student status may capture a combination of sustained reading, intensive near work, prolonged indoor activity, and frequent electronic-device use. However, work status is a broad social and occupational indicator rather than a direct measure of visual exposure. Work status may reflect differences in educational demand, daily activity, and other unmeasured exposures rather than an inherently protective effect of specific occupations.

Longer electronic-device use and shorter sleep duration were associated with myopia, and these variables also showed associations in selected severity models, particularly the high-astigmatism model. Previous systematic reviews and meta-analyses have reported associations among near work, screen time, sleep characteristics, and myopia-related outcomes (35–37). Contemporary refractive epidemiological models suggest that cumulative lifestyle exposures, including educational demands, digital engagement, and reduced sleep, may interact with refractive development (38). After multivariable adjustment, device use of 5–8 hours/day and >8 hours/day was associated with higher odds of myopia than 1–4 hours/day, while sleep duration ≤6 hours was also associated with higher odds. This pattern is compatible with an exposure-response association, but temporality and causality cannot be established. Device duration and sleep were self-reported, and the study did not distinguish educational from recreational screen use, continuous from interrupted viewing, viewing distance, or time spent outdoors. Complementary evidence from Iraqi university students linked longer screen exposure to greater ocular symptom burden, although dry-eye symptoms and refractive status are distinct outcomes (39).

A positive family history of refractive error emerged as one of the strongest independent predictors of myopia in this study, highlighting the importance of familial susceptibility. This finding is consistent with evidence that parental myopia and familial aggregation are associated with refractive outcomes (40). Genetic predisposition may also interact with environmental exposures, including near work and reduced outdoor time (41, 42). The persistence of this association after multivariable adjustment supports inclusion of family history in clinical risk assessment (13). Nevertheless, family history was self-reported as a binary variable, without information on the number of affected relatives, parental refractive magnitude, or shared household behaviors. The observed association may therefore represent both inherited susceptibility and shared environmental exposure.

Severity-specific analyses suggested distinct association patterns for high myopia, high hyperopia, and high astigmatism. These models provide information beyond the primary myopia analysis, but they should be considered exploratory. Precision was limited for high hyperopia because of the smaller number of events, and model discrimination was limited for high myopia (AUC = 0.593) and modest for high hyperopia (AUC = 0.667). In addition, multiple comparisons were performed without multiplicity adjustment, increasing the possibility of chance findings. Confirmation is needed in larger population-based studies using ocular biometry, cycloplegic refraction where appropriate, and longitudinal follow-up.

The strengths of this study include its large sample size, multicenter design, standardized refractive-assessment protocol, use of one eye per participant, and evaluation of prespecified demographic, social, familial, and behavioral variables. The use of right-eye data ensured statistical independence in accordance with established methodological recommendations in ophthalmic epidemiological research (43). The analysis used the relevant sex- and age-group denominators and refractive outcomes derived from standardized sphere and cylinder measurements. Consecutive recruitment from three tertiary ophthalmic centers yielded a large multicenter clinical sample.

These findings provide a large multicenter clinical profile of refractive errors among young adults attending tertiary ophthalmic services in Iraq. The identified associations with sex, family history, sleep duration, electronic-device exposure, and work or study status may assist targeted history taking, refractive assessment, counseling, and planning of services for young adults in similar clinical settings.

Several limitations should be acknowledged. The cross-sectional design limits causal inference, and behavioral variables were self-reported and may be affected by recall or reporting bias. The absence of cycloplegic refraction may have underestimated hyperopia and shifted spherical-equivalent measurements toward myopia, particularly among younger participants (12, 28). The hospital-based sampling frame limits generalizability: participants were tertiary-clinic attendees and should not be considered representative of all Iraqi young adults. The marked female predominance (70.0%) may reflect sex-related differences in clinic attendance and further limits generalizability. Referral patterns, symptoms, access to services, and care-seeking behavior may also have influenced the observed distributions. The source dataset lacked a prescreening log, so the number assessed before construction of the analytic file and reasons for non-inclusion could not be reconstructed. Important potential confounders, including time outdoors, educational intensity, near-work distance, continuous task duration, socioeconomic circumstances, and parental refractive magnitude, were not measured in sufficient detail. The severity-specific analyses involved multiple comparisons without multiplicity adjustment, and the high-myopia and high-hyperopia models had limited-to-modest discrimination. Finally, analysis of the right eye ensured independence but did not assess inter-eye asymmetry or classify participants according to the more severely affected eye.

Despite these limitations, this multicenter study provides clinically relevant evidence on refractive outcomes among young adults attending ophthalmic centers in Iraq. The findings support risk-based counseling that considers family history, sleep duration, electronic-device exposure, and educational or occupational context, without implying population-level prevalence or causality. Future community-based longitudinal studies should incorporate cycloplegic refraction, ocular biometry, and standardized measures of near work and outdoor exposure. Counseling should also address safe contact lens use and regular corneal assessment, given reported endothelial changes with prolonged wear in Iraqi young adults (44). For eligible myopic adults, individualized selection of laser refractive procedures is warranted because available techniques differ in predictability, stability, visual quality, and ocular-surface outcomes (45).

## Conclusions

Among young adults attending three tertiary ophthalmic centers in Iraq, myopia and astigmatism were frequent, and myopia showed independent associations with sex, family history, sleep duration, electronic-device exposure, and work or study status. These multicenter data provide a clinically useful profile that may support targeted assessment, counseling, and planning of refractive services. Population-based longitudinal studies are needed to determine the generalizability and temporal nature of these associations.

## Data Availability

The raw data supporting the conclusions of this article will be made available by the authors, without undue reservation.
